# IL-27 Limits Type 2 Immunopathology Following Parainfluenza Virus Infection

**DOI:** 10.1371/journal.ppat.1006173

**Published:** 2017-01-27

**Authors:** Gaia Muallem, Sagie Wagage, Yan Sun, Jonathan H. DeLong, Alex Valenzuela, David A. Christian, Gretchen Harms Pritchard, Qun Fang, Elizabeth L. Buza, Deepika Jain, M. Merle Elloso, Carolina B. López, Christopher A. Hunter

**Affiliations:** 1 Department of Pathobiology, School of Veterinary Medicine, University of Pennsylvania, Philadelphia, Pennsylvania, United States of America; 2 Department of Nephrology, University of Pennsylvania, Philadelphia, Pennsylvania, United States of America; 3 Department of Immunobiology, Yale University School of Medicine, New Haven, Connecticut, United States of America; 4 Janssen Research & Development, LLC, Immunology Discovery Research, Spring House, Pennsylvania, United States of America; University of North Carolina at Chapel Hill, UNITED STATES

## Abstract

Respiratory paramyxoviruses are important causes of morbidity and mortality, particularly of infants and the elderly. In humans, a T helper (Th)2-biased immune response to these infections is associated with increased disease severity; however, little is known about the endogenous regulators of these responses that may be manipulated to ameliorate pathology. IL-27, a cytokine that regulates Th2 responses, is produced in the lungs during parainfluenza infection, but its role in disease pathogenesis is unknown. To determine whether IL-27 limits the development of pathogenic Th2 responses during paramyxovirus infection, IL-27-deficient or control mice were infected with the murine parainfluenza virus Sendai virus (SeV). Infected IL-27-deficient mice experienced increased weight loss, more severe lung lesions, and decreased survival compared to controls. IL-27 deficiency led to increased pulmonary eosinophils, alternatively activated macrophages (AAMs), and the emergence of Th2 responses. In control mice, IL-27 induced a population of IFN-γ^+^/IL-10^+^ CD4^+^ T cells that was replaced by IFN-γ^+^/IL-17^+^ and IFN-γ^+^/IL-13^+^ CD4^+^ T cells in IL-27-deficient mice. CD4^+^ T cell depletion in IL-27-deficient mice attenuated weight loss and decreased AAMs. Elimination of STAT6 signaling in IL-27-deficient mice reduced Th2 responses and decreased disease severity. These data indicate that endogenous IL-27 limits pathology during parainfluenza virus infection by regulating the quality of CD4^+^ T cell responses and therefore may have therapeutic potential in paramyxovirus infections.

## Author Summary

Respiratory viral infections are important propagators of acute and chronic disease, and a subset of those affected require hospitalization. Type 2 immune responses have a well-established association with increased disease severity; however, these responses have not been manipulated to ameliorate disease severity in clinical practice. Here we show that the cytokine interleukin-27 (IL-27) limits immunopathology after paramyxovirus infection. IL-27 regulates the quality of the inflammatory response, independent of viral replication, by restricting pathologic CD4^+^ T cell- and type 2 innate immune responses. As such, IL-27 emerges as an endogenous regulator of pathologic inflammation after respiratory viral infection and therefore may have both diagnostic and therapeutic potential in clinical medicine.

## Introduction

Acute respiratory infections are an important cause of morbidity and mortality in children and adults [1, 2]. Paramyxoviruses including respiratory syncytial virus (RSV), parainfluenza virus, and metapneumovirus are among the most frequent causes of severe illness [1]. Although type 1 immune responses characterized by the generation of Th1 CD4^+^ T cells are essential for viral clearance and the development of protective immunity during these infections, severity of illness is associated with type 2 polarization of the immune response [3–5]. Despite their clinical importance, the mechanisms that regulate the development of type 2 immune responses during respiratory viral infections are unknown.

IL-27 is a heterodimeric cytokine composed of the Epstein-Barr virus-induced gene 3 (EBi3, also shared with IL-35) and IL-27p28 subunits [6, 7]. It engages a receptor formed by gp130 and the IL-27Rα to activate the Janus kinase (JAK)-signal transducer and activator of transcription (STAT) signaling pathway [6, 8]. Endogenous IL-27 regulates T cell responses in various models of inflammation [9–12]. In addition to antagonizing T cell production of IL-2, IL-27 directly inhibits Th2 and Th17 activities [10, 11, 13] and is a potent inducer of IL-10, a cytokine that antagonizes the function of antigen presenting cells (APC) [14, 15].

The murine parainfluenza virus Sendai (SeV) induces an acute respiratory infection in mice that is cleared by the immune system and that leads to type 2 immune pathology in the lung at chronic time points, mimicking what is observed in humans [16, 17]. IL-27p28 transcripts are increased transiently in the lungs of mice during SeV infection [17], and IL-27 is required for the expansion of Sendai-specific CD8^+^ T cells [18, 19]; however, the cellular source of IL-27 and its effects on viral control and on the development of immune pathology remain unknown.

Here we show that following infection with SeV, IL-27 deficiency leads to increased lung pathology and disease severity as well as to higher frequencies of eosinophils and alternatively activated macrophages (AAMs), consistent with an increased bias towards a type 2 immune response. SeV infection in control mice induced IFN-γ from CD4^+^ T cells as well as a subset of IL-10-producing IFN-γ^+^ CD4^+^ T cells in the lung. IL-27 deficiency was associated with a loss of the IFN-γ/IL-10 double producers and with the emergence of IFN-γ/IL-13 and IFN-γ/IL-17 double-producing CD4^+^ T cells. Depletion of CD4^+^ T cells in IL-27Rα^-/-^ mice led to a decrease in AAMs and reduced weight loss after infection, while eliminating STAT6 signaling in IL-27-deficient mice reduced Th2 responses and decreased mortality. Taken together, these studies support a model in which IL-27 promotes IFN-γ^+^/IL-10^+^ CD4^+^ T cells and restricts the emergence of pathologic CD4^+^ T cells that contribute to type 2 immune pathology during Sendai virus infection.

## Results

### SeV infection induces transient production of IL-27p28 by lung macrophages

**Fig 1:**
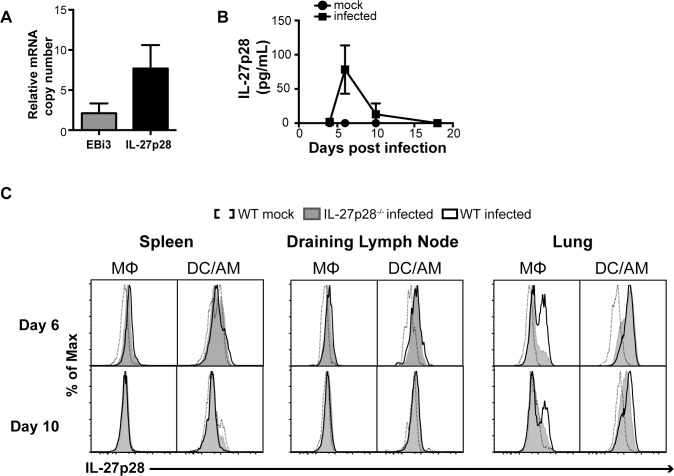
IL-27 is produced in the lung in response to SeV. Female WT mice were infected intranasally with SeV. **(A)** After 10 days, total lung tissue was homogenized, and RT PCR was performed to evaluate expression of EBi3 and IL-27p28. Data are representative of three independent experiments with three to five mice per group **(B)** BALF was evaluated by ELISA to quantify IL-27p28 protein at the time points indicated. Data are representative of three mice per group **(C)** Cells from the spleen, dLN, and lung were analyzed for IL-27p28 expression by FACS. Populations shown were gated on live cells negative for CD3, CD19, and NK1.1. DC/AM are CD11c^hi^, MHC II^+^. MΦ are CD11c^int^, MHC II^-^. As indicated, data points are representative of 6 and 10 dpi and are representative of two independent experiments with three mice per group.

Sendai virus replication in the lung has been associated with increased levels of IL-27p28 mRNA [17], but the cellular source was unclear. In mice, IL-27p28 is secreted in excess, and EBi3 is secreted when co-expressed with IL-27p28 [20]. Accordingly, at 10 days post infection (dpi) transcripts of IL-27p28 were detected in higher amounts than EBi3 by qPCR relative to internal control (Fig 1A). Consistent with these findings, analysis of bronchoalveolar lavage (BAL) protein revealed elevated IL-27p28 production at 6 and 10 dpi with no IL-27p28 detected at 4 and 18 dpi (Fig 1B). To identify the sources of IL-27, different populations of APCs in the lungs and secondary lymphoid organs of mock- or SeV-infected C57BL/6 wild type (WT) mice were evaluated by flow cytometry for IL-27p28 expression. Given the high levels of monocyte autofluorescence in the lung, infected IL-27p28^-/-^ mice were also used as a control. Analysis of lung, draining lymph nodes, and spleen revealed that IL-27p28 was not detected in mock controls at local or peripheral sites or in infected mice at 4 or 22 dpi. No IL-27p28 was detected in the spleen or draining lymph nodes (dLN) (Fig 1C) at any time point after infection. In the lung, IL-27p28 was primarily produced by a population of CD11c^mid^MHC II^-^ cells (Fig 1C). These cells were CD11b^+^ and expressed high levels of Ly6c and CD64, consistent with monocytes and/or monocyte-derived macrophages (S1 Fig). In the absence of CCR2, a population of CD11c^mid^MHC II^-^/CD11b^+^/CD64^+^/Ly6c^-^ cells produced IL-27 in response to SeV, suggesting that other lung macrophages retain the ability to make IL-27 even in the absence of recruited monocytes (S1 Fig). No IL-27p28 was detected from CD11c^hi^MHC II^+^ cells, representative of dendritic cells (DCs) and/or alveolar macrophages (AM). Neither CD4^+^ nor CD8^+^ T cells produced IL-27p28. These data indicate that a discrete population of lung macrophages transiently produces IL-27 in the lung after SeV.

### Loss of IL-27 signaling is associated with increased disease severity but not with increased viral load during SeV infection

**Fig 2:**
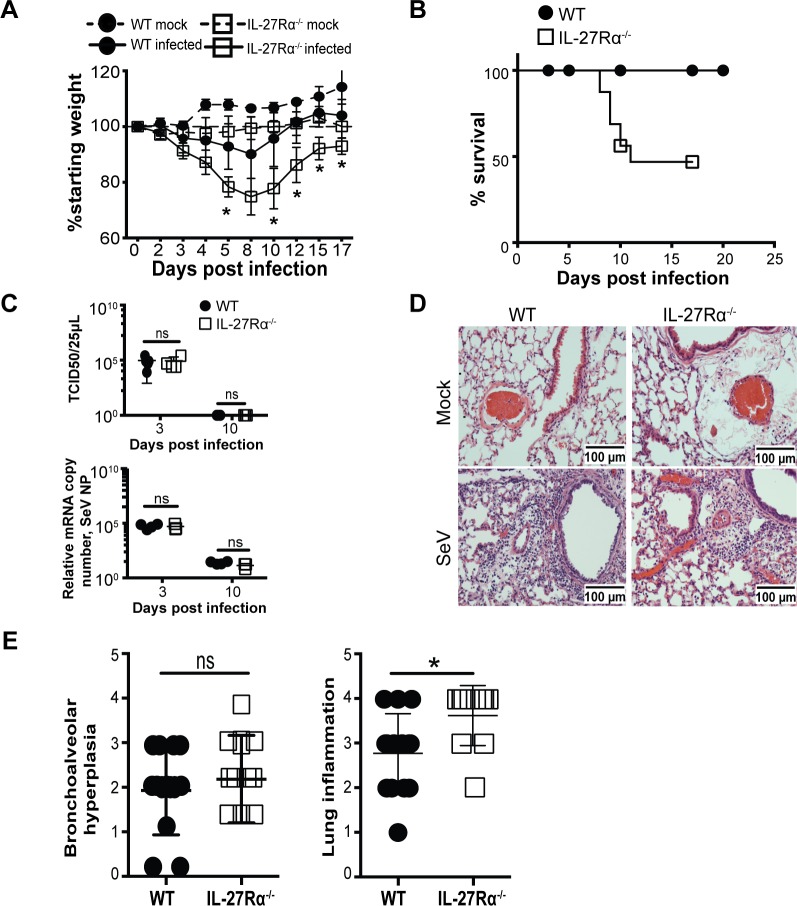
Loss of IL-27 signaling results in increased disease severity and lung immunopathology after SeV. **(A)** Female mice were infected intranasally with SeV and weighed every two days until 17 dpi. Weights are graphed as percent of starting weight. Plotted weights are of two mice per group (mock) and five mice per group (infected) and are representative of weight trends in five separate experiments *(p<0*.*0001)*. **(B)** Survival of infected males after SeV. Findings are pooled from five experiments and include WT (n = 25) and IL-27Rα^-/-^ (n = 18) mice *(p* = *0*.*0009*, Mantel-Cox test) **(C)** Whole lung tissue from infected females was homogenized, and viral load was quantified by titration of infected cells and RT-PCR at 3 and 10 dpi. Data are representative of two separate experiments, three to five mice per group. **(D)** Sections of lung tissue from WT and IL-27Rα^-/-^ mice were preserved in formalin and stained by hematoxylin and eosin (H&E) at 10 dpi. Mock indicates intranasal inoculation with PBS (vehicle) **(E)** Lung pathology was scored for severity of inflammation. Histological scores are pooled from three separate experiments with three to five mice per group (*p<0.*0155*, Mann-Whitney test).

To directly assess the impact of endogenous IL-27 on the outcome of SeV infection, WT and IL-27Rα^-/-^ mice were monitored for weight loss as a measure of disease progression. Infected WT mice experienced transient weight loss; however, consistent with enhanced disease severity, infected IL-27Rα^-/-^ mice lost more weight than controls (Fig 2A). The increased weight loss observed in IL-27Rα^-/-^ mice was consistent across multiple experiments using male or female mice, and similar results were observed using IL-27p28^-/-^ and EBi3^-/-^ mice. Male gender is associated with increased severity of respiratory diseases in mice and humans [21], and in fitting with this increased severity of disease half of the infected male mice succumbed to infection between days 9 to 11 (Fig 2B). To determine whether the increased morbidity observed in IL-27Rα^-/-^ mice was due to a reduced ability to clear virus, viral load was evaluated at the peak of SeV replication (3 dpi) and at the time of viral clearance (10 dpi) [22]. Infected WT and IL-27Rα^-/-^ mice had comparable viral titers and levels of viral transcripts (Fig 2C) at these time points. These data indicate that the increased disease severity seen in the IL-27Rα^-/-^ mice was not due to impaired viral clearance.

To determine if the absence of the IL-27Rα was associated with more severe lung pathology, WT and IL-27Rα^-/-^ mice were infected, and lung tissue was analyzed for histopathological differences at 10 dpi. Mice given a mock infection with intranasal PBS did not show any evidence of lung inflammation (Fig 2D). In infected mice, inflammatory lesions were consistent with published reports and included multifocal bronchointerstitial pneumonia, alveolar and bronchiolar epithelial necrosis, and inflammatory cell infiltrate composed of lymphocytes, plasma cells, neutrophils, and macrophages [22]. Although bronchoalveolar hyperplasia was not different between the groups, in comparison to infected WT mice, infected IL-27Rα^-/-^ mice exhibited more severe inflammation in the lung, although to a lesser degree than expected given the differences in weight loss and survival (Fig 2D and 2E). Despite the increased mortality in males, no obvious differences in viral clearance, lung lesions, or inflammatory cell types of interest were observed between males and females when compared at 10 dpi (S2 Fig).

### IL-27 limits type 2 innate immune responses in the lung during SeV infection

**Fig 3:**
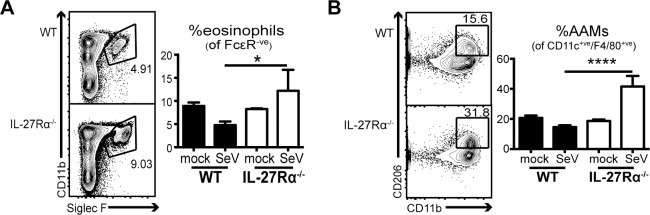
IL-27 limits type 2 innate immune responses in the lung after SeV infection. Mice were infected intranasally with SeV and control PBS (mock), and lungs were harvested and evaluated for cellular infiltrate at 10 dpi. Frequency of **(A)** pulmonary eosinophils (CD3^-^/CD19^-^/NK1.1^-^/FcεR^-^/CD11b^+^/Siglec F^+^), and **(B)** alternatively activated macrophages by flow cytometry (CD3^-^/CD19^-^/NK1.1^-^/CD45^+^/CD11c^+^/CD11b^+^/CD206^+^). For AAMs the same results were obtained using the marker CD64 in place of CD11c. Flow plots are of infected mice using groups of male or female mice. Plots are of three to five mice per group and are representative of six experiments (*p<0.*05*, ****p<0.*0001*).

Type 2 immune responses are important mediators of lung pathology at late time points after SeV infection [16, 17]; however, this phenotype has not been described in the acute phase of the immune response to the virus. Flow cytometric analysis of granulocyte populations at 10 dpi in IL-27Rα^-/-^ mice showed an increased frequency and number of eosinophils in the lung (Fig 3A, S4 Fig), which is unexpected after SeV. In addition, there was a corresponding increase in the frequency and number of CD11b^+^ macrophages expressing the mannose receptor (CD206^+^) (Fig 3B, S4 Fig), a marker of AAMs [23]. Of note, the increases in eosinophils and AAMs were consistent among mice deficient in each subunit of IL-27 (EBi3^-/-^, IL-27p28^-/-^) (S3 Fig). Moreover, the shift to a type 2 immune response in the lung was consistent among infected males and females (S2 Fig). Therefore, while SeV is associated with a Th1-type response in the lungs of WT mice [22, 24], the data presented here suggest that the increased disease severity observed in IL-27Rα^-/-^ mice may be associated with an immune environment more consistent with Th2-like responses.

### IL-27 regulates polyfunctional CD4^+^ T cell populations during SeV infection

**Fig 4:**
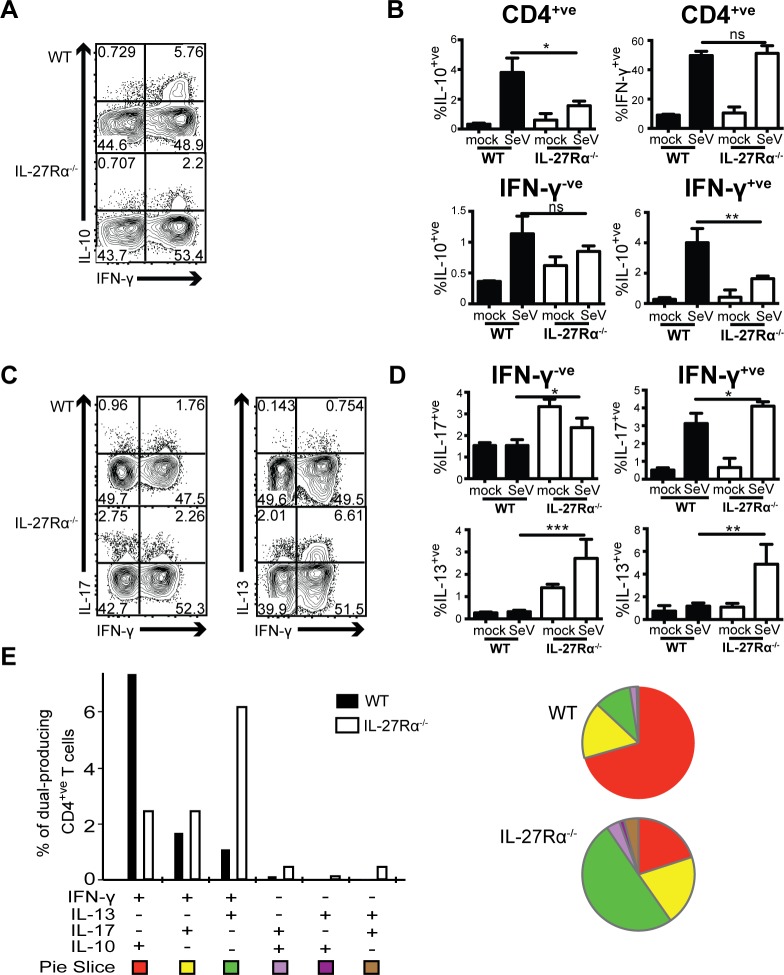
Loss of IFN-γ^+^/IL-10^+^ CD4^+^ T cells in IL-27 deficiency is associated with the emergence of Th2 and Th17 responses. WT and IL-27Rα-/- mice were infected intranasally with SeV or control PBS (mock). Lung lymphocytes were isolated and restimulated ex vivo with PMA/ionomycin/BFA/monensin for 4 hours at 10 dpi, then stained intracellularly for cytokine production. Data shown are representative of five experiments, three to five mice per group. **(A)** Flow plots and **(B)** graphical representation of lung CD4^+^ T cells after restimulation for IL-10 and IFN-γ (above). Frequency of IL-10 production by IFN-γ^+ve^ and IFN-γ^-ve^ CD4^+^ T cells (below). **(C)** Flow plots showing IL-13 and IL-17 production as they relate to IFN-γ production by CD4^+^ T cells. **(D)** Frequency of IL-13 and IL-17 production by IFN-γ^+ve^ and IFN-γ^-ve^ CD4^+^ T cells. **(E)** SPICE analysis representation of CD4^+^ T cell production of IL-13, IL-10, IFN-γ, IL-17 in all cells producing two cytokines. Flow plots are of infected female mice. (*p<0.*05*, **p<0.*01*, ***p = 0.*001*).

Previous studies have highlighted that during SeV infection IL-27 promotes the expansion of Sendai-specific CD8^+^ T cells in the lung and that it induces IL-10 production by CD8^+^ T cells *in vitro* and *in vivo* [15, 18]. We also observed a decreased frequency of virus-specific CD8^+^ T cells in IL-27Rα^-/-^ mice during SeV infection but did not detect a difference in IL-10 production by the total CD8^+^ T cell population (S5 Fig). However, CD4^+^ T cells are the major cytokine-producing T cells in the lung after SeV [22, 25], and the regulatory role of IL-27 on CD4^+^ T cells has not been explored in this model. In the absence of IL-27, CD4^+^ T cells from the lungs of mice infected with SeV produced less IL-10 at 10 dpi (Fig 4A and 4B). CD4^+^ T cells also represent a major source of IFN-γ after SeV infection, with peak production occurring at 10 dpi [22]. Therefore, lymphocytes were isolated from infected lungs at 10 dpi and stimulated *ex vivo* with phosphomolybdic acid (PMA), ionomycin, B-refeldin, and monensin to evaluate cytokine production by the total CD4^+^ T cell population. At this time point, total IFN-γ production by all CD4^+^ T cells was comparable in WT and IL-27Rα^-/-^ mice and therefore independent of IL-27 signaling (Fig 4A and 4B). However, although total levels of IFN-γ were unchanged in infected IL-27Rα^-/-^ mice, a population of the total CD4^+^ T cells that was double positive for IFN-γ and IL-10 was reduced in frequency (Fig 4A and 4B) and number (S4 Fig), consistent with work in other models of inflammation [11, 15, 26]. IL-27 also directly suppresses IL-4/IL-13 and IL-17 synthesis by CD4^+^ T cells [10, 27], and although levels of these cytokines in the BAL were not different between groups, analysis of all CD4^+^ T cells from infected IL-27Rα^-/-^ mice revealed marked increases in IL-17 and IL-13 production (Fig 4C and 4D, S4 Fig). There was also a significant increase in the frequency of IFN-γ^+^ CD4^+^ T cells that co-produced IL-13 or IL-17 (Fig 4C and 4D). Of note, these data were consistent among mice deficient in each subunit of IL-27 (EBi3^-/-^, IL-27p28^-/-^) as well as in the IL-27Rα^-/-^ mice (S3 Fig). Together, these results suggest that after SeV not only does IL-27 limit Th2 and Th17 responses in the lung but that it also prevents the development of polyfunctional IFN-γ^+^/IL-13^+^ and IFN-γ^+^/IL-17^+^ CD4^+^ T cells in this context.

Next, the SPICE data mining software was used to analyze the polyfunctional CD4^+^ T cell populations and evaluate for phenotypic patterns in infected WT and IL-27Rα^-/-^ mice. As expected, the IFN-γ^+^/IL-10^+^ CD4^+^ T cells were the predominant polyfunctional population in infected WT mice (Fig 4E). In infected IL-27Rα^-/-^ mice, the reduction in IFN-γ^+^/IL-10^+^ CD4^+^ T cells was associated with the emergence of both IFN-γ^+^/IL-17^+^ and IFN-γ^+^/IL-13^+^ CD4^+^ T cells. However, these populations were not present in the lung at equal frequencies. Consistent with the type 2 innate immune cells found in the lungs of IL-27Rα^-/-^ mice, IFN-γ^+^/IL-13^+^ CD4^+^ T cells represented the largest population of dual producers (Fig 4E). Of the cytokines examined, neither WT nor IL-27Rα^-/-^ CD4^+^ T cells were producing more than two cytokines concurrently. From these data, we conclude that during SeV infection IL-27 production induces a population of IFN-γ^+^/IL-10^+^ CD4^+^ T cells and that the loss of IL-27 signaling leads to the emergence of IFN-γ^+^/IL-17^+^ and IFN-γ^+^/IL-13^+^ polyfunctional CD4^+^ T cells.

### IL-27 limits pathologic Th2 responses during SeV

**Fig 5:**
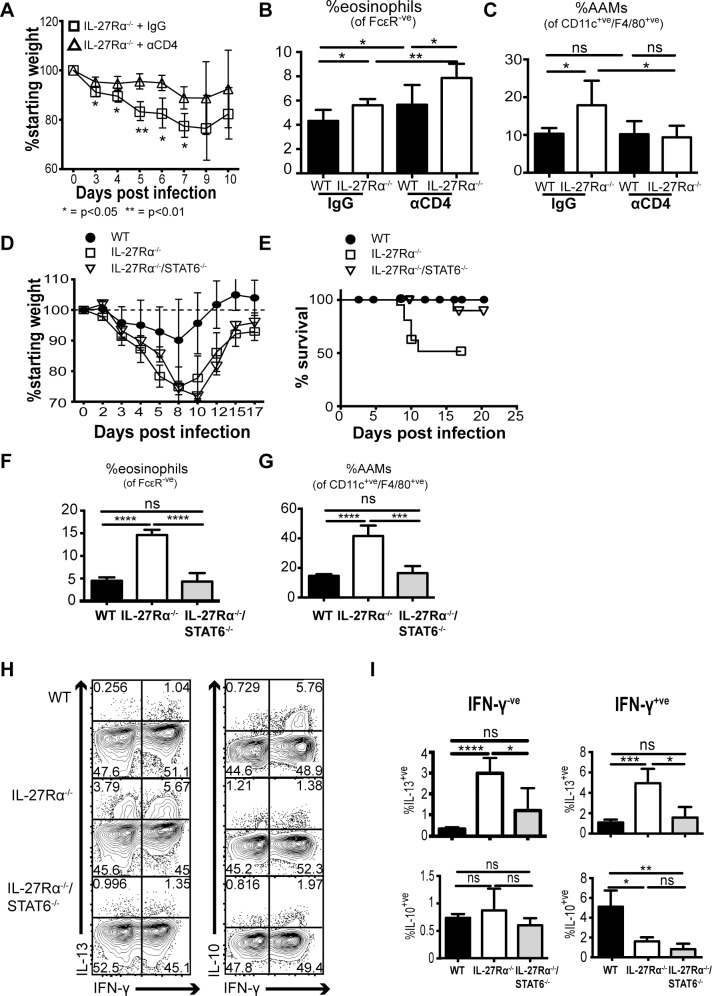
Pathogenic Th2 responses emerge in the absence of IL-27 (A-D). WT and IL-27Rα^-/-^ mice were infected with SeV, and monoclonal anti-CD4 antibody versus isotype control was administered at 0, 4, and 7 dpi (data representative of two experiments, three to five mice per group) **(A)** Weight change in IL-27Rα^-/-^ plus isotype versus IL-27Rα^-/-^ plus anti-CD4. **(B-C)** Lung lymphocytes were isolated at 10 dpi and evaluated by flow cytometry for frequency of **(B)** eosinophils, and **(C)** AAMs. **(D-E)** WT, IL-27Rα^-/-^, and IL-27Rα^-/-^/STAT6^-/-^ mice were infected with SeV (data representative of three experiments, three to five mice per group). **(D)** Weight loss plotted over time in each group (infected mice) **(E)** Percent survival of infected male mice over time **(F-G)** Lung tissue was taken for analysis at 10 dpi with frequencies of **(F)** eosinophils, and **(G)** AAMs plotted. **(H-I)** Lung lymphocytes were isolated and restimulated ex vivo with PMA/ionomycin/BFA/monensin for 4 hours, then stained intracellularly for cytokine production. **(H)** Flow cytometric and **(I)** Graphical representation of cytokine production by lung CD4^+^ T cells after restimulation for IFN-γ, IL-10, and IL-13. Representative plots are from male mice. (*p<0.*05*, **p<0.*01*, ***p<0.*001*, ****p<0.*0001*).

Since the IL-27Rα^-/-^ mice infected with SeV develop more severe disease that correlates with an altered CD4^+^ T cell phenotype, we next asked whether CD4^+^ T cells contributed to the enhanced morbidity. In these experiments, infected mice were treated with isotype control or a monoclonal antibody to deplete CD4^+^ T cells before and during SeV infection. Of note, the depletion of CD4^+^ T cells in WT mice did not abrogate the infection-induced changes in weight (S6 Fig), suggesting that the CD4^+^ T cells in WT mice are not pathogenic. In contrast, while infected isotype-treated IL-27Rα^-/-^ mice lost weight as expected, depletion of CD4^+^ T cells dramatically decreased this weight loss (Fig 5A). CD4^+^ T cell depletion did not abrogate the pulmonary eosinophilia in IL-27Rα^-/-^ mice (Fig 5B), but the frequency of AAMs in these mice was reduced to baseline levels after CD4^+^ T cell depletion (Fig 5C). Taken together, these data indicate that the absence of the IL-27Rα after SeV infection results in CD4^+^ T cells that have pathologic effects associated with an increase in AAMs.

Corresponding to the differences seen in infected IL-27Rα^-/-^ mice, Th2-like responses are associated with enhanced lung damage in murine and human respiratory infection [3, 5]. Thus, experiments were performed to assess the impact of IL-4/IL-13 signaling on disease severity in IL-27Rα^-/-^ mice. Because STAT6 is critical for IL-4- and IL-13-mediated signaling [28], mice deficient in the IL-27Rα and STAT6 (IL-27Rα^-/-^/STAT6^-/-^) were generated and then challenged with SeV. As previously shown, WT mice had transient weight loss, but IL-27Rα^-/-^ mice experienced more severe weight loss after SeV. IL-27Rα^-/-^/STAT6^-/-^ mice also lost more weight than WT mice but did not show any improvement over IL-27Rα^-/-^ mice (Fig 5D). However, the increased mortality seen in infected IL-27Rα^-/-^ male mice was ameliorated by elimination of STAT6 signaling (Fig 5E). As expected, the loss of STAT6 signaling in the IL-27Rα^-/-^ mice resulted in a marked decrease in frequency of pulmonary eosinophils (Fig 5F) and AAMs (Fig 5G), and these mice did not mount Th2 responses after SeV (Fig 5H and 5I, S4 Fig). However, IL-27Rα^-/-^/STAT6^-/-^ mice did exhibit a persistent loss of IFN-γ^+^/IL-10^+^ CD4^+^ T cells (Fig 5H and 5I), and IL-17 production by IFN-γ^-^ and IFN-γ^+^ CD4^+^ T cells was also unchanged (S7 Fig). Therefore, elimination of STAT6 signaling reduced Th2 responses in IL-27Rα^-/-^ mice and mitigated disease severity without restoring the IFN-γ^+^/IL-10^+^ CD4^+^ T cells. Together, these results suggest that IL-27 inhibits the development of Th2 responses during the acute response to SeV infection and that in the context of IL-27 deficiency the emergence of these responses contribute to the T cell-mediated pathology.

## Discussion

The data presented here reveal that IL-27Rα^-/-^ mice challenged with SeV experienced increased disease severity mediated by CD4^+^ T cells as well as type 2 innate immune responses. In multiple models of infection the loss of the IL-27Rα has been associated with CD4^+^ T cell-mediated immunopathology linked with the overproduction of IFN-γ [12, 29, 30]. While the ability of CD4^+^ T cell depletion to ameliorate disease in IL-27Rα^-/-^ mice is similar to other systems [12, 31], the contribution of the STAT6-dependent Th2 response to this pathology is unique and indicates that IL-27 is an important endogenous regulator of Th2 immune responses during parainfluenza virus infection.

There are multiple potential sources of endogenous IL-27, and while macrophages and DCs are considered major contributors [32], a more recent report suggested that during infection with *Plasmodium berghei* CD4^+^ T cells also produced IL-27 [33]. While we found no evidence that T cells produced IL-27p28 during this infection, there was a distinct population of monocytes and/or monocyte-derived macrophages in the lungs that produced IL-27p28. The induction of IL-27 during this infection could be a direct response to SeV [32] or may represent a secondary response to the SeV-induced production of type I interferons [32, 34] as during experimental autoimmune encephalitis and multiple sclerosis, the ability of type I IFNs to promote IL-27 is linked to their clinical efficacy [35].

Interestingly, SeV infection is lethal in the majority of male IL-27Rα^-/-^ mice but not in females. Males have been shown to exhibit increased disease severity in both clinical and preclinical studies of respiratory illness [21, 36], and in other inflammatory systems immune responses are influenced by gender [37, 38]. An improved understanding of the impact of gender on immune responses during respiratory viral infection could have important implications for risk stratification and disease management in humans. Although no differences were noted between the immune parameters evaluated in males and females in this study, the increased mortality in male mice deficient in IL-27 signaling presents a unique opportunity for further study.

Based on work from our laboratory and others, abrogating type 2 immunity after SeV infection is not only important for limiting disease severity at early time points but also affects disease progression in the chronic phase [16, 17]. Interestingly, while CD4^+^ T cell depletion and elimination of STAT6 signaling in infected IL-27Rα^-/-^ mice both led to decreased disease severity and fewer AAMs, these two interventions did not phenocopy each other. Pulmonary eosinophilia was decreased in infected IL-27Rα^-/-^/STAT6^-/-^ mice but was unchanged by CD4^+^ T cell depletion. This sustained eosinophilia in the absence of CD4^+^ T cells may be explained by the ability of IL-27 to inhibit proliferation and cytokine production by type 2 innate lymphoid cells (ILC2s) [39, 40]. It could also be explained by epithelial cells, which express the IL-27Rα [41] and whose production of IL-13 contributes to airway remodeling [42]. Although the emergence of Th2-like innate and adaptive cells was the predominant phenotype in infected IL-27Rα^-/-^ mice, Th17 cells have also been linked to immunopathology in IL-27Rα^-/-^ mice infected with respiratory syncytial virus (RSV) [43], and our data did show persistent Th17 responses in infected IL-27Rα^-/-^/STAT6^-/-^ mice. This suggests that Th17 cells may contribute to the immunopathology seen during SeV infection in IL-27 deficiency. Ultimately, despite the notable differences, both CD4^+^ T cell depletion and elimination of STAT6 signaling abrogated disease severity in infected IL-27Rα^-/-^ mice. Therefore, by limiting type 2-mediated pathology after SeV, IL-27 regulates a clinically relevant pathway of the immune response after parainfluenza virus infection.

The production of IL-10 is an important mechanism to limit many types of inflammatory processes [11, 15, 29], and several cytokines including IL-6, IL-12, IL-21, IL-27, and type I interferons have been shown to promote T cell secretion of IL-10 [15, 44]. Many of these cytokines are produced during SeV, but the loss of the IFN-γ^+^/IL-10^+^ double producers in the IL-27Rα^-/-^ mice indicates a dominant role for IL-27 in this experimental system. Until recently CD4^+^ T cell subsets were defined largely on a restricted ability to make unique profiles of cytokines (Th1 making IFN-γ, Th2 making IL-4/IL-13, Th17 making IL-17) after lineage commitment. However, co-expression of these cytokines can occur, and our data are reminiscent of studies that have shown human CD4^+^ T cells specific for *C*. *albicans* and *M*. *tuberculosis* that produce IFN-γ also possess the capacity to make IL-4 or IL-17 [45]. Dual cytokine-producing CD4^+^ T cells also have been identified in models of autoimmunity and viral infection [46–48], and IFN-γ^+^/IL-17^+^ CD4^+^ T cells have been described in pathogenic post viral immune responses [49]. The impact of IL-27 on these dual producers has not been previously described but is consistent with the ability of IL-27 to directly limit Th2 and Th17 differentiation [10, 11] and may represent a mechanism to limit T cell heterogeneity during viral infection.

The experiments presented here suggest that while the T cell response to SeV infection is dominated by the production of IFN-γ, IL-27 acts on CD4^+^ T cells not only to promote the production of IL-10 but also to limit the emergence of pathologic CD4^+^ T cell responses associated with severe disease. Identifying an endogenous regulator of type 2 immune responses in the lung during parainfluenza virus has therapeutic implications. Genetic analyses have established connections between *IL-27p28* polymorphisms and the severity of acute respiratory infection in premature infants [50] and of chronic lung disease in adults and children [51–53], but additional clinical studies are needed to look at this relationship in the context of respiratory viral infection. Nevertheless, taken together with the data presented here, these studies indicate that endogenous IL-27 may have a broad role in limiting immunopathology in the respiratory system and may be useful as an adjunct therapy to limit disease progression.

## Methods

### Mice

EBi3^-/-^, and IL-27p28^-/-^ mice were provided by Sage Research Labs (Boyertown, PA). IL-27Rα^-/-^ mice were provided by Amgen and bred in our facility. C57BL/6 mice were from Sage Research Labs (Boyertown, PA) or Taconic (Germantown, NY). STAT6^-/-^ mice were obtained from The Jackson Laboratory (Bar Harbor, ME). All mice strains were housed, maintained, and bred under specific pathogen-free conditions at the University of Pennsylvania.

### Ethics statement

All experimental procedures with mice were approved by the Institutional Animal Care and Use Committee (IACUC) of the University of Pennsylvania in accordance with guidelines of the Association for Assessment and Accreditation of Laboratory Animal Care (Protocol #805045). IACUC uses the NIH Guide for the Care and Use of Laboratory Animals, which is based on the U.S. Government Principles for Utilization and Care of Vertebrate Animals Used in Testing, Research, and Training. Mice were euthanized by administration of CO_2_ for at least five minutes in accordance with these guidelines.

### Viruses and infection

SeV strain 52 was propagated in the laboratory of C.B.L. as previously described [34]. Mice were infected intranasally with 10 ID_50_ of SeV strain 52 (10,000 TCID_50_/mouse) after anesthesia with ketamine and xylazine. Mice were infected after anesthesia with ketamine and xylazine. Mice were weighed on the day of infection (day 0) and every two days following, with weight loss calculated as percent change from original weight. Virus titration was performed by hemagglutination of chicken red blood cells (Lampire Biological Products) as previously described [34]. Briefly, the lungs were extracted, homogenized in PBS-gelatin (1%), and frozen at -80°C for preservation. The presence of infectious particles was evaluated by infecting LLCMK2 cells with 1:10 dilutions of the lung homogenates at 37°C. After 1 hour of infection, 175 μL of medium containing 2 μg/mL trypsin was added and the cells were further incubated for 72h at 37°C. A total of 25μL of medium was then removed from the plate and tested by hemagglutination of chicken red blood cells (RBCs) for the presence of virus particles. Viruses at 1:4 dilutions in 0.5% chicken RBCs were incubated for 30 min at 4°C. The hemagglutination of RBCs indicated the presence of virus particles [24]. For the CD4^+^ T cell depletion, anti-CD4 monoclonal antibody (clone GK1.5, BioXCell) was administered at 250 μg/mouse on days 0, 4, 7. Rat IgG2b (BioXcell) was used as an isotype control.

### Real-time quantitative PCR

Total RNA was purified from lungs using TRIzol (Life Technologies). RNA was reverse transcribed and amplified with specific primers in the presence of Power SYBR Green PCR Master Mixture (Applied Biosystems; Life Technologies). The primers for IL-27p28, EBi3, and β-actin were obtained from Quantitect (QIAGEN), and the SeV *Np* primer sequence was as follows: (Forward *3’*-TGCCCTGGAAGATGAGTTAG Reverse *5’*-GCCTGTTGGTTTGTGGTAAG). Normalization was conducted based on levels of beta-actin.

### Cell preparation and analysis

Single cell suspensions were generated from mouse spleens, lungs, and lymph nodes. Mouse spleens and lymph nodes were collected and dissociate through a 70 μm strainer. Red blood cells from spleens were lysed using 0.85% ammonium chloride (Sigma). Red blood cell lysis was not performed on lymph nodes. Lungs were inflated with a solution of 1 mg/ml collagenase A (Roche) and 100 μg/ml DNase (Roche), and then diced and digested in the same solution for 60 min at 37°C to obtain a single-cell suspension. Red blood cells were lysed using 0.86% ammonium chloride (Sigma). Cells from all tissues were counted, washed in flow cytometry buffer (1% BSA (Sigma), 2mM EDTA (Invitrogen) in PBS) and stained for surface markers. For assessment of cytokine production, T cells were restimulated with PMA and ionomycin plus brefeldin A (BGA) and monensin (Sigma) and stained for surface markers, then fixed with 4% paraformaldehyde for ten minutes prior to intracellular staining for relevant cytokines [54]. Cells were blocked with 2.4G2 (BioXCell) and Rat IgG (Invitrogen) before staining with monoclonal antibodies. Samples were acquired using an LSRFortessa flow cytometer (BD Biosciences) and analyzed with FlowJo software (Tree Star, Inc.) and SPICE software (NIAID). Viable cells were identified using the LIVE/DEAD Fixable Aqua Dead Cell Stain Kit for 405nm excitation (Invitrogen). The following mAb against mouse antigens were used for staining: FITC-anti-CD3 (clone 145-2c11), FITC-anti-CD19 (clone 6D5), FITC-anti-NK1.1 (clone PK136), APC-anti-FcεR (clone MAR-1), PE-anti-Siglec F (clone E50-2440), ef780-anti-CD11b (clone M1/70), PerCP-anti-Ly6c (clone HK1.4), Pacific Blue-anti-Ly6G (clone 1A8), ef780-anti-CD3 (clone 145-2c11), PE CF594-anti-CD4 (clone GK1.5), Pacific Blue-anti-CD8α (clone 53–6.7), FITC-anti-Foxp3 (clone 150D/E4), PE Cy7-anti-IFN-γ (clone XMG1.2), PE-anti-IL-13 (clone eBio13A), APC-anti-IL-10 (clone JES5-16E3), PerCP-anti-IL-17A (clone eBio17B7), AF700-anti-CD45 (clone 30-F11), ef780-anti-CD11c (clone N418), PE-anti-F4/80 (clone BM8), PerCP-anti-CD11b (clone M1/70), APC-anti-CD206 (clone C068C2), PE-anti-CD11a (clone H155-78), PE-Cy7-anti-KLRG1 (clone 2F1), Pacific Blue-anti-MHC class II (clone M5/114.15.2). IL-27p28 production was evaluated in the lung by flow cytometry (APC-anti-IL-27p28, clone MM27-7B1) and in the bronchoalveolar lavage fluid using an IL-27p28 specific ELISA (R&D Systems). Antigen-specific cells were identified using a conjugated tetramer to the Sendai virus nucleoprotein fragment FAGPNYPAL, provided by the NIH tetramer core. IL-27p28 production was evaluated in the lung by flow cytometry and in the bronchoalveolar lavage fluid using an IL-27p28 specific ELISA (R&D Systems).

### Histologic assessment of lung inflammation

After lavage, the left lobe of the lung was inflated and fixed with 0.5 ml of 10% neutral-buffered formalin solution. Fixed lung tissues were embedded in paraffin, and sections were cut using a standard procedure. Deparaffinized sections from fixed lungs were stained with hematoxylin and eosin. Lung inflammation was scored by a board-certified veterinary pathologist (E.L.B.). Lung inflammation was scored according to the following scale: 0, no manifestation; 1, minimal and peribronchiolar only; 2, mild, in areas of epithelial hyperplasia; 3, moderate; and 4, severe. The severity of bronchiolar/alveolar epithelial hyperplasia was scored as percent of affected 40X area and percent of terminal bronchioles affected, as follows: Percent of affected area: 0, 0%; 1, 1–25%; 2, 26–50%; 3, 51–75%; 4, 76–100%; Percent of terminal bronchioles affected: 0, 0–20%; 1, 21–40%; 2, 41–60%; 3, 61–80%; 4, 81–100%. Bronchiolar smooth muscle hyperplasia was evaluated as follows: 0, none; 1, mild; 2, moderate; 3, severe.

### Statistics

Bar graphs and scatter plots were plotted as means with the SEM in Prism 5 software (GraphPad). All statistics were performed using an unpaired Student *t* test, except the histologic evaluation between WT, IL-27Rα^-/-^, and IL-27Rα^-/-^/STAT6^-/-^ in which the Mann-Whitney test was used and survival analyses, which were evaluated by the log-rank (Mantel-Cox) test.

## Supporting Information

S1 Fig Cells making IL-27p28 express Ly6c and CD64. Gating strategy and expression markers of IL-27p28 positive verses negative cells in the lungs of mock and SeV infected mice at 10 dpi after stimulation with monensin and BFA for 6 hours prior to staining.
(TIF)

S2 Fig Female and male mice have comparable immune phenotypes after SeV. Viral titers by qRT-PCR, lung inflammation scoring, and immune cell characteristics in infected females versus male mice at 10 dpi. Cytokine production by CD4^+^ T cells was determined by intracellular staining after restimulation for 4 hours with PMA/ionomycin/BFA/monensin.
(TIF)

S3 Fig Consistent immune responses in IL-27 subunit knockout mice. WT, EBi3^-/-^, and IL-27p28^-/-^ mice were infected with SeV, and cellular responses were characterized for frequency of eosinophils, AAMs, and CD4^+^ T cell responses at 10 dpi. After restimulation for 4 hours with PMA/ionomycin/BFA/monensin, CD4^+^ T cells were evaluated for production of IFN-γ, IL-10, IL-13, and IL-17.
(TIF)

S4 Fig Enumeration of cells infiltrating lungs of WT versus IL-27Rα^-/-^ mice. Innate and adaptive immune responses were assessed in WT and IL-27Rα^-/-^ mice at 10 dpi. Cytokine production by CD4^+^ T cells was determined by intracellular staining after restimulation for 4 hours with PMA/ionomycin/BFA/monensin.
(TIF)

S5 Fig IL-27 promotes CD8^+^ T cell responses during SeV infection. WT and IL-27Rα^-/-^ mice were infected, and antigen-specific CD8^+^ T cells in lung lymphycoytes were evaluated by flow cytometry at 10 dpi. Cytokine production by CD8^+^ T cells was determined by intracellular staining after restimulation for 4 hours with PMA/ionomycin/BFA/monensin.
(TIF)

S6 Fig CD4^+^ T cell depletion does not abrogate weight loss in infected WT mice. WT mice were infected with SeV, and monoclonal anti-CD4 antibody versus isotype control was administered at 0, 4, and 7 dpi. Weight change of infected WT mice + isotype versus infected WT mice + anti-CD4 is shown.
(TIF)

S7 Fig Th17 responses remain intact in infected IL-27Rα^-/-^/STAT6^-/-^ mice. WT, IL-27Rα^-/-^, and IL-27Rα^-/-^/STAT6^-/-^ mice were infected with SeV. Lung lymphocytes were isolated at 10 dpi and restimulated for 4 hours with PMA/ionomycin/BFA/monensin before evaluation by flow cytometry for intracellular cytokine production. Shown are flow cytometric and graphical representation of CD4^+^ T cell production of IFN-γ and IL-17.
(TIF)
