# Supplementary figures and images for: IL-27 Limits Type 2 Immunopathology Following Parainfluenza Virus Infection
Source: PLoS Pathog. 2017 Jan 27;13(1):e1006173. doi: 10.1371/journal.ppat.1006173 (PMC5305264; doi:10.1371/journal.ppat.1006173)

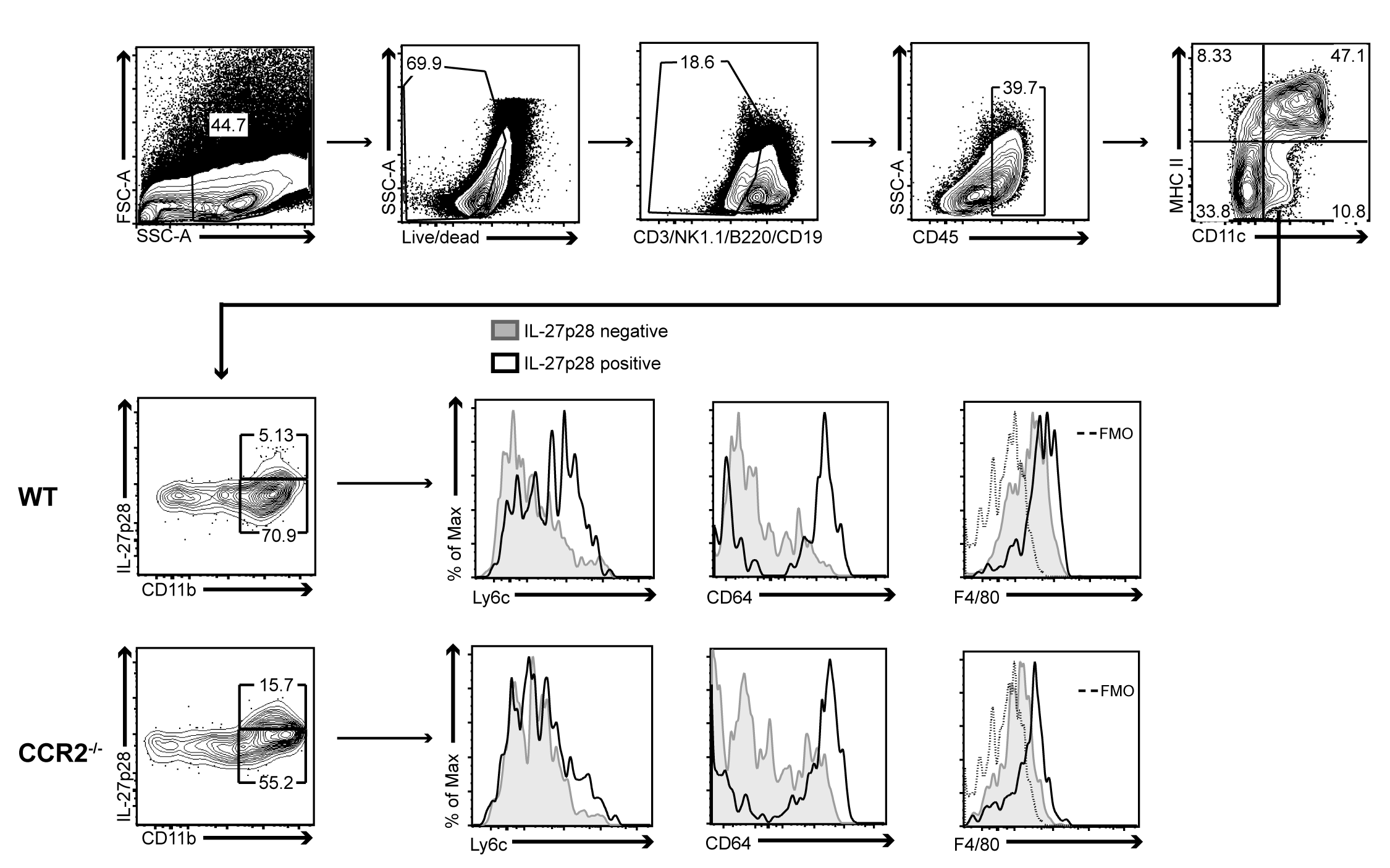

Supplement: S1 Fig — Gating strategy and expression markers of IL-27p28 positive verses negative cells in the lungs of mock and SeV infected mice at 10 dpi after stimulation with monensin and BFA for 6 hours prior to staining. (TIF) [file ppat.1006173.s001.tif]

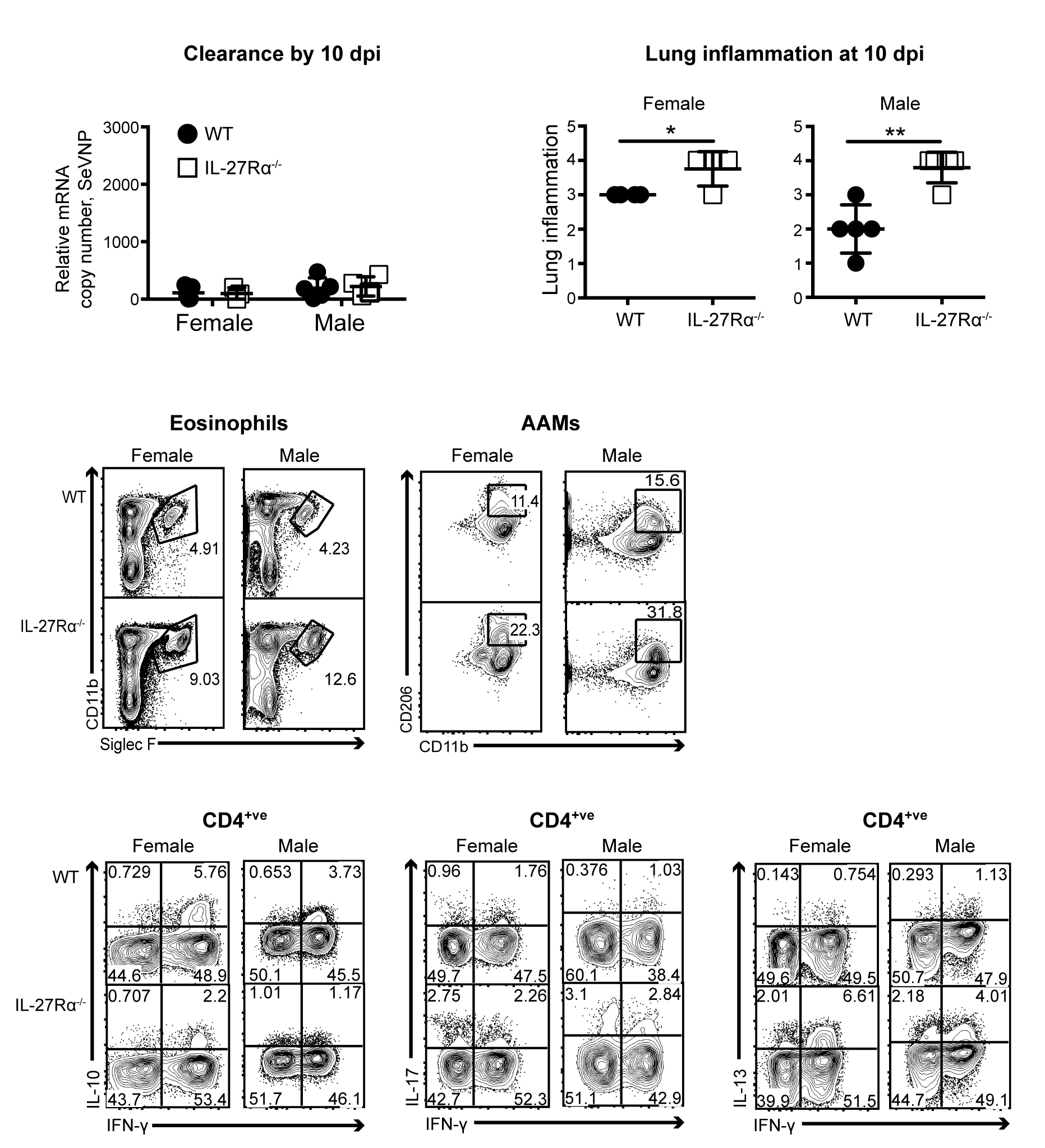

Supplement: S2 Fig — Viral titers by qRT-PCR, lung inflammation scoring, and immune cell characteristics in infected females versus male mice at 10 dpi. Cytokine production by CD4+ T cells was determined by intracellular staining after restimulation for 4 hours with PMA/ionomycin/BFA/monensin. (TIF) [file ppat.1006173.s002.tif]

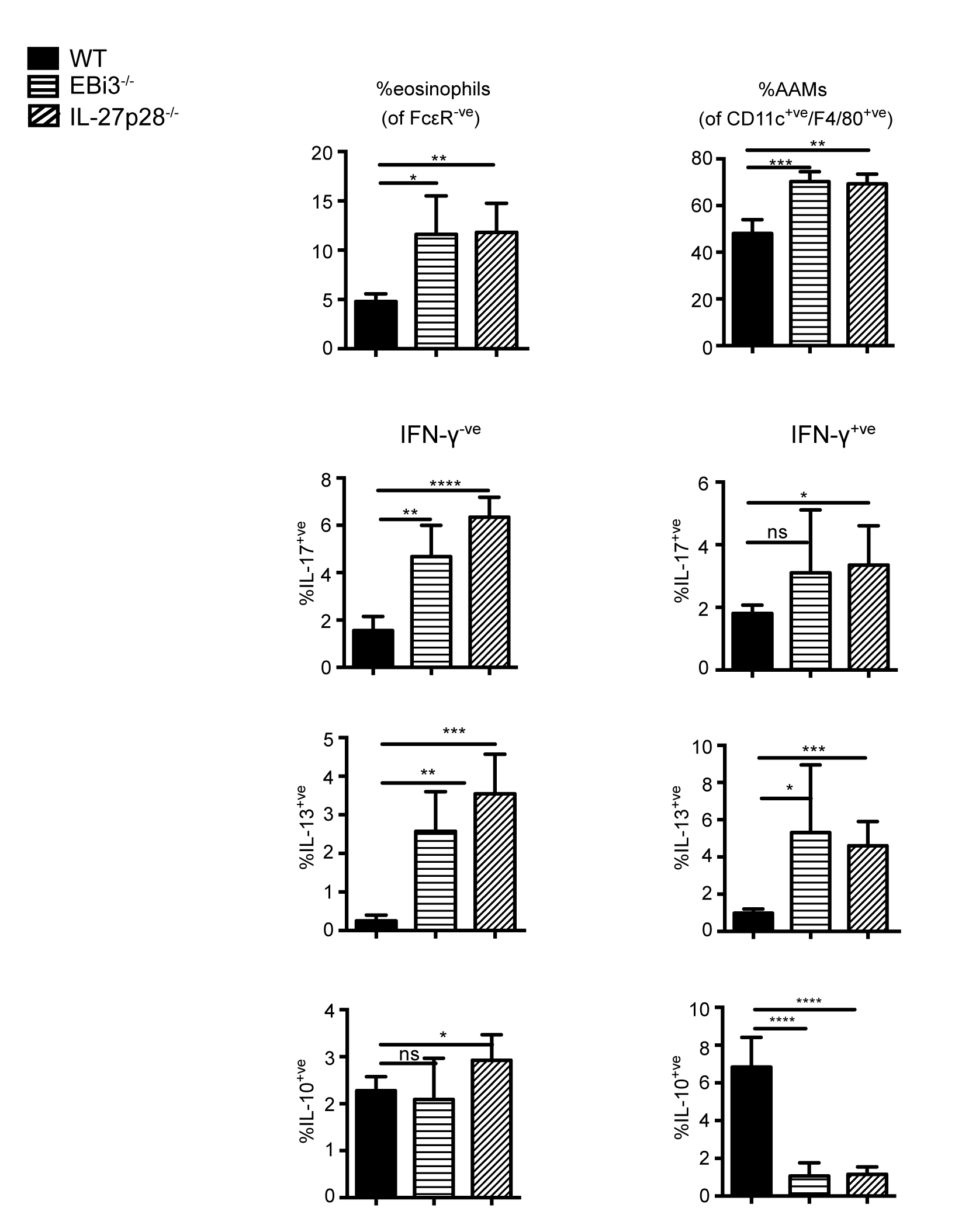

Supplement: S3 Fig — WT, EBi3-/-, and IL-27p28-/- mice were infected with SeV, and cellular responses were characterized for frequency of eosinophils, AAMs, and CD4+ T cell responses at 10 dpi. After restimulation for 4 hours with PMA/ionomycin/BFA/monensin, CD4+ T cells were evaluated for production of IFN-γ, IL-10, IL-13, and IL-17. (TIF) [file ppat.1006173.s003.tif]

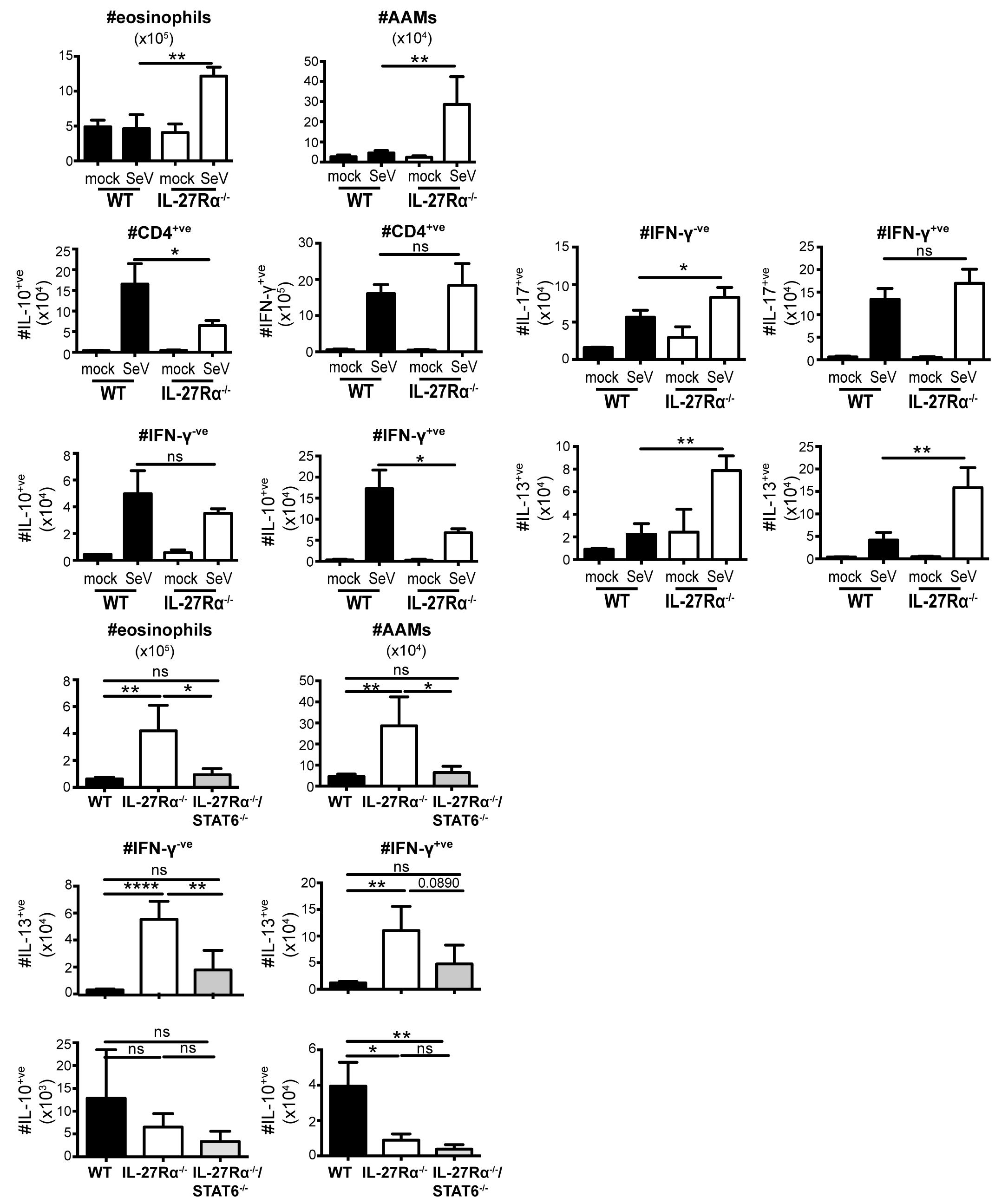

Supplement: S4 Fig — Innate and adaptive immune responses were assessed in WT and IL-27Rα-/- mice at 10 dpi. Cytokine production by CD4+ T cells was determined by intracellular staining after restimulation for 4 hours with PMA/ionomycin/BFA/monensin. (TIF) [file ppat.1006173.s004.tif]

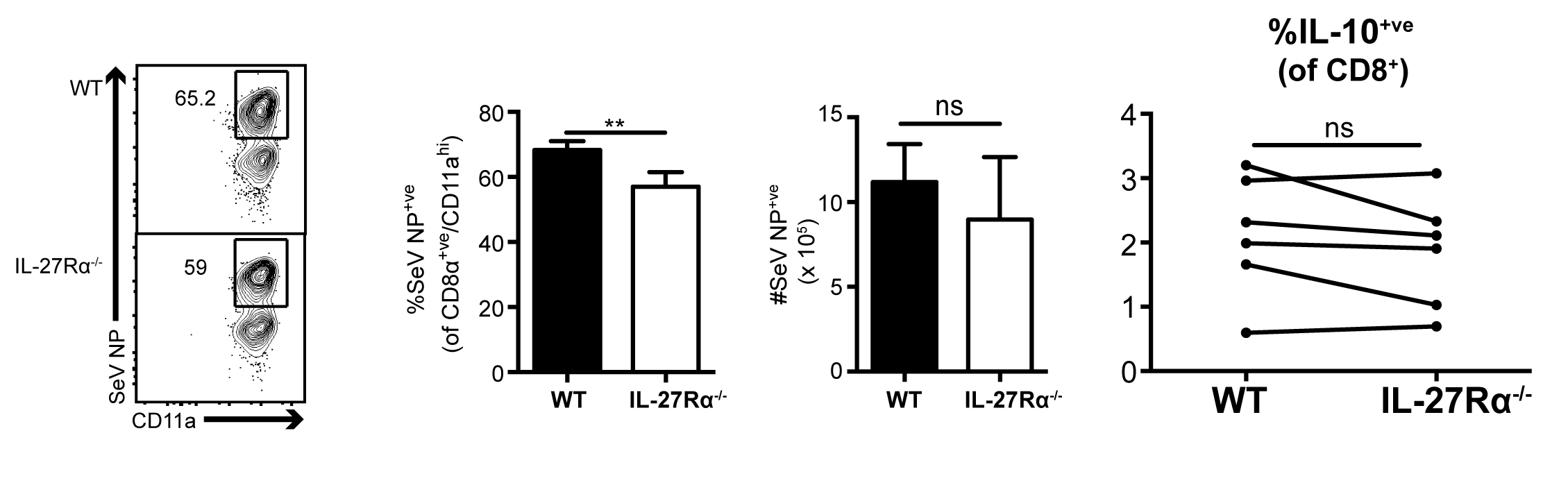

Supplement: S5 Fig — WT and IL-27Rα-/- mice were infected, and antigen-specific CD8+ T cells in lung lymphycoytes were evaluated by flow cytometry at 10 dpi. Cytokine production by CD8+ T cells was determined by intracellular staining after restimulation for 4 hours with PMA/ionomycin/BFA/monensin. (TIF) [file ppat.1006173.s005.tif]

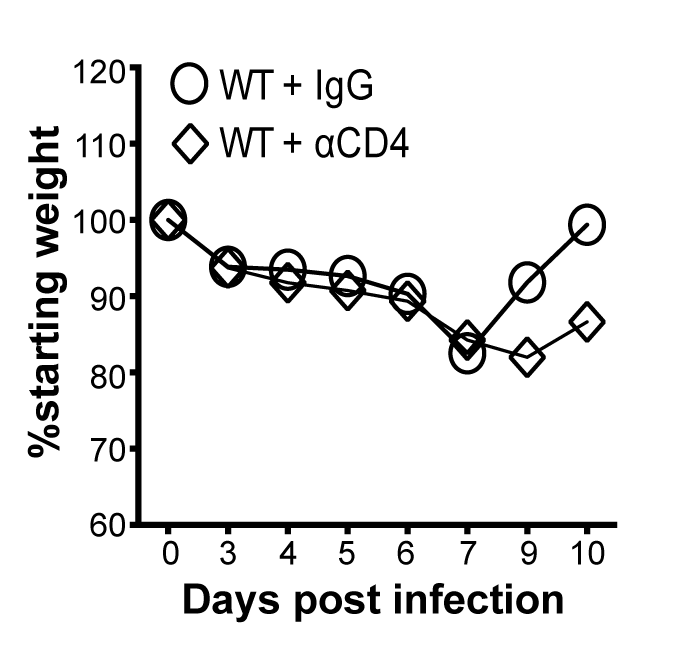

Supplement: S6 Fig — WT mice were infected with SeV, and monoclonal anti-CD4 antibody versus isotype control was administered at 0, 4, and 7 dpi. Weight change of infected WT mice + isotype versus infected WT mice + anti-CD4 is shown. (TIF) [file ppat.1006173.s006.tif]

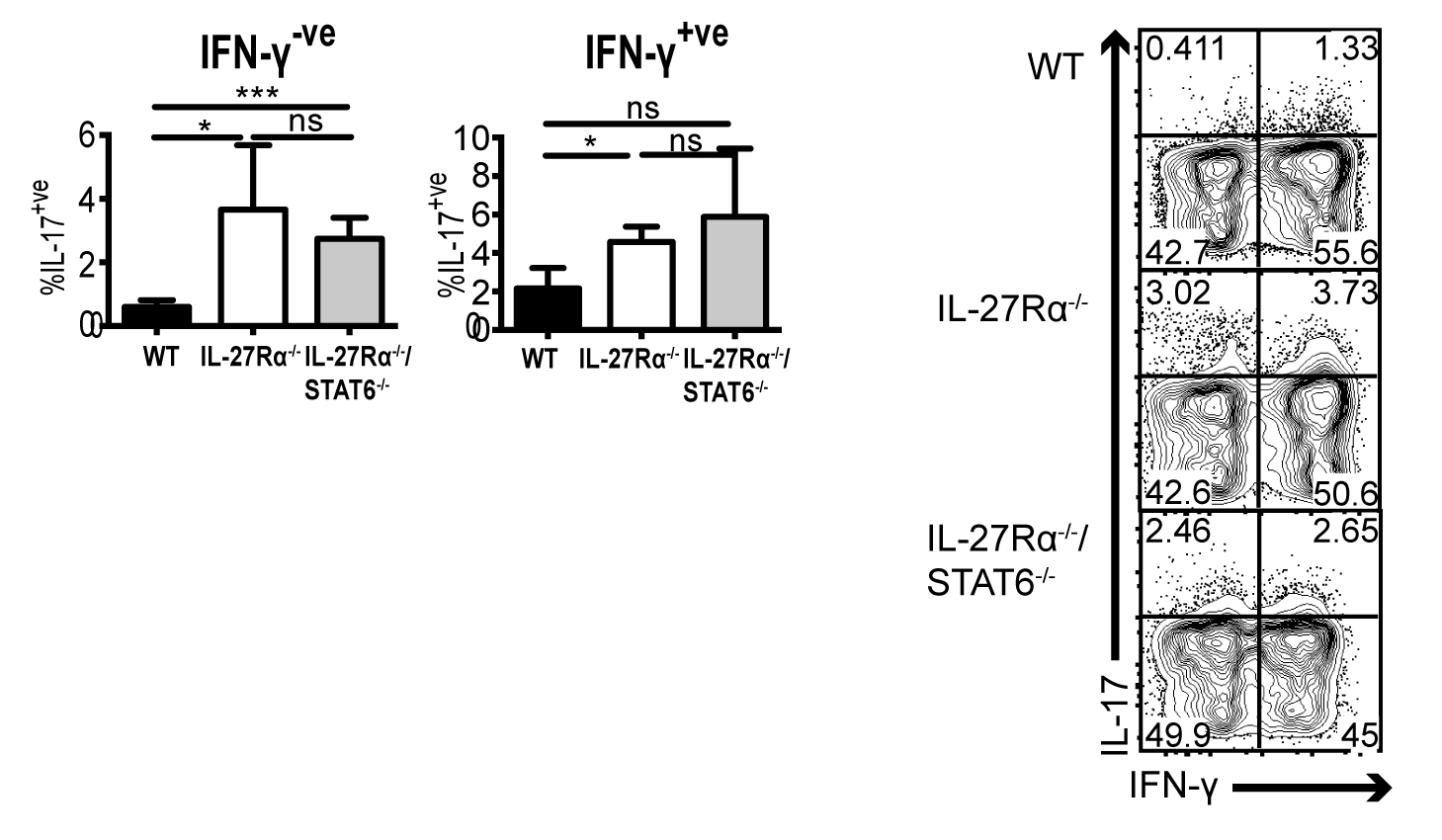

Supplement: S7 Fig — WT, IL-27Rα-/-, and IL-27Rα-/-/STAT6-/- mice were infected with SeV. Lung lymphocytes were isolated at 10 dpi and restimulated for 4 hours with PMA/ionomycin/BFA/monensin before evaluation by flow cytometry for intracellular cytokine production. Shown are flow cytometric and graphical representation of CD4+ T cell production of IFN-γ and IL-17. (TIF) [file ppat.1006173.s007.tif]
